# Identification of Novel TAT-I24-Related Peptides with Antiviral Activities

**DOI:** 10.3390/ijms262311433

**Published:** 2025-11-26

**Authors:** Hanna Harant, Siegfried Höfinger, Reingard Grabherr, Zsolt Ruzsics, Hartmut Hengel

**Affiliations:** 1 Pivaris BioScience GmbH, Media Quarter Marx 3.4, Maria-Jacobi-Gasse 1, 1030 Vienna, Austria; 2 VSC Research Center, TU Wien, 1040 Vienna, Austria; siegfried.hoefinger@tuwien.ac.at; 3 Department of Physics, Michigan Technological University, Houghton, MI 49931, USA; 4 Institute for Molecular Biotechnology, Department for Biotechnology and Food Science, BOKU University, Muthgasse 18, 1190 Vienna, Austria; reingard.grabherr@boku.ac.at; 5 Institute of Virology, Medical Center-University of Freiburg, 79104 Freiburg, Germany; zsolt.ruzsics@uniklinik-freiburg.de (Z.R.); hartmut.hengel@uniklinik-freiburg.de (H.H.); 6 Faculty of Medicine, University of Freiburg, 79110 Freiburg, Germany

**Keywords:** antiviral peptide, antimicrobial peptide, mouse cytomegalovirus, baculovirus, molecular modelling

## Abstract

To identify novel peptides with potential antiviral activities, a database search was performed based on the primary sequence of the peptide I24 (CLAFYACFC), the effective part of the antiviral peptide TAT-I24 consisting of peptide I24 and the cell penetrating TAT-peptide (amino-acids 48–60; GRKKRRQRRRPPQ). A Protein BLAST search identified several sequences with high similarity to I24 in diverse proteins, some of which are known to be involved in the interaction with nucleic acids. Selected sequences and newly designed variants of I24 were synthesized as TAT fusion peptides and tested for antiviral activity in two well-established models: baculovirus transduction of HEK293 cells and mouse cytomegalovirus (MCMV) infection of NIH/3T3 cells. Several of the TAT-fusion peptides exhibited antiviral activities with a potency comparable to TAT-I24. The ability of these peptides to bind double-stranded DNA suggested the same mode of action. Several peptides caused swelling of red blood cells (RBC) but with only one peptide clearly inducing haemolysis. With two exceptions, RBC swelling was observed with antivirally active peptides but not with less active peptides, indicating that antiviral activities are linked to an effect on membrane integrity of target cells. Structural prediction of the TAT-fusion peptides indicated formation of two α-helical elements, with several of these peptides showing remarkable similarity when subjected to structural alignment.

## 1. Introduction

Emerging viral infections and the increasing prevalence of antibiotic-resistant bacteria pose a threat to modern societies and require the urgent development of novel treatments [1,2]. Apart from low molecular weight chemical compounds, antimicrobial peptides (AMPs) have emerged as promising alternative antimicrobial compounds [3,4,5,6]. However, the use of peptide drugs is still limited by their low stability, short half-lives and low bioavailability by oral application, which can be overcome by modifying the peptides and delivery systems [5,7,8].

AMPs are produced by virtually all organisms and are used as primary defence mechanisms against pathogens [3,9,10]. In vertebrates, in particular, frogs are a rich source of AMPs [11]. Mammals also produce AMPs, such as defensins [12,13,14] and cathelicidins, such as the human cathelicidin LL-37 [15,16]. Another peptide with antimicrobial activity is the liver-derived cysteine-rich 25-mer peptide hepcidin [17]. Most AMPs act on lipid bilayers and disrupt membrane integrity of bacteria or enveloped viruses. However, AMPs can also act intracellularly on various targets, such as by interaction with nucleic acids as described for defensins [18] or frog-derived peptides such as buforin II [19,20] and pseudin-2 [21], as well as the bovine AMP indolicidin [22] and fish-derived piscidins [23]. AMPs share specific physico-chemical properties, including small size, the presence of hydrophobic and cationic amino-acid residues resulting in a positive net charge and frequently amphipathic nature [9,10]. In addition, their secondary structure is important but can undergo dynamic changes depending on their environment, such as membranes [9]. Antiviral peptides (AVP) are a subset of AMPs, several of which can act against both bacteria and viruses via membrane or intracellular targets [24].

We have developed and characterized the synthetic antiviral peptide TAT-I24, which can neutralize a variety of double-stranded (ds) DNA viruses, including herpes simplex viruses, cytomegaloviruses, adenoviruses, vaccinia virus and SV40 polyoma virus [25]. The peptide binds dsDNA with high affinity and can thereby inhibit viral gene expression [26]. TAT-I24 is also inhibiting replication of the RNA virus SARS-CoV-2, although its antiviral activity is dependent on the viral entry route as differential sensitivities to the peptide were observed for SARS-CoV-2 variants [27]. A similar activity pattern was also observed with various adenovirus serotypes, indicating that the viral entry route is one determining factor for its activity [25,27].

We therefore aimed to identify novel peptides similar to TAT-I24 with potentially improved features and antiviral activities. For this, we used a literature and sequence database search to identify similar segments in known proteins. We could retrieve several peptide sequences with similarities to TAT-I24 in various proteins and synthesized, based on these sequences, small peptides as TAT-fusions. Several of the peptides exhibited potent antiviral activity with a related mode-of-action to TAT-I24.

## 2. Results

### 2.1. Identification of Novel Sequences Conferring Antiviral Activity

The peptide TAT-I24 is a fusion of the cell-penetrating TAT-peptide (amino acids 48–60 of the HIV TAT-protein; [28,29]) and the 9-mer peptide I24 (CLAFYACFC) and has been shown to neutralize a variety of viruses in vitro [25,27]. The antiviral effect is mediated via the I24 peptide, while the TAT peptide facilitates cell attachment and entry of the fusion peptide. Furthermore, the TAT peptide greatly enhances the DNA-binding affinity of I24 [26]. To identify peptides with a high similarity to the 9-mer peptide I24, a sequence search by Protein BLAST [30] using the primary amino-acid sequence CLAFYACFC as the query was conducted [31]. In addition, a search for peptides with a lower similarity to I24 but containing similar types of amino acids was also performed. Finally, literature and database searches for similar sequence motifs in known proteins with DNA-binding properties were performed. Table 1 shows the results from the Protein BLAST search and selection of synthetic sequences for subsequent experimental analysis.

Peptides containing novel sequences based on the original I24 sequence were also generated by rational design with the aim to improve the features and antiviral potency of the peptide by deleting or replacing cysteine residues or duplication of the LAFYA-motif, such as peptide M40 (LAFYACLAF), which lacks the C-terminal residue of peptide M14, peptide M219 (LAFYASLAF), which has a replacement of cysteine 6 of M40, and peptides M212 (CLAFYACLAFYAC) and M214 (YLAFYALAFYAC).

**Figure 1:**
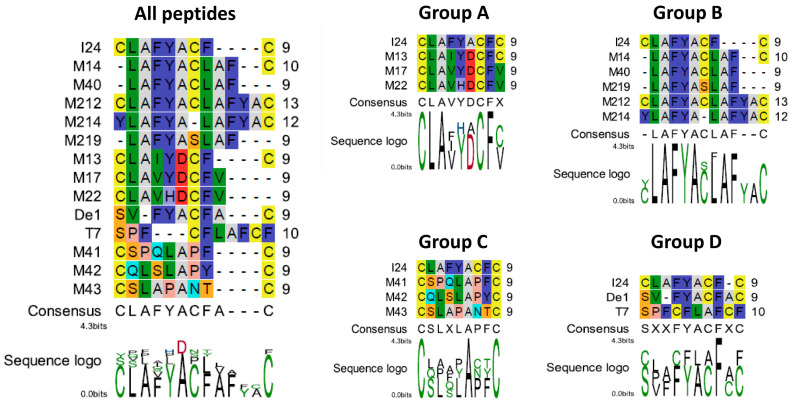
Alignment of peptide sequences and association to different groups. Alignment of all peptides are shown on the left. Group A represents an alignment of I24 with sequences found in the Nsp14 protein of beta-coronaviruses, group B shows an alignment of I24 with sequences containing the LAFYA motif, group C shows an alignment of I24 with sequences less similar to I24 and group D shows an alignment of I24 with De1, a synthetic sequence, and T7, a sequence found in T7RNAP. Amino acids are shown in Rasmol colours; the sequence logo is shown in polarity colours.

Sequences were then aligned using the CLC Biobench, as shown in Figure 1. Based on these alignments, peptides were assigned to different groups. Group A represents peptide sequences found in the N-terminal domain with exonuclease activity (ExoN domain) of the Nsp14 protein of beta-coronaviruses. The closest match with I24 was obtained with peptide M13 derived from the Nsp14 protein of the beta-coronavirus HKU1 (amino acids 276–284 of the Nsp14 protein). Sequences with higher differences to the I24 query sequence were peptides M17 (derived from Nsp14 protein of bat *betacoronavirus* sp.) and M22 (derived from Nsp14 protein of SARS-CoV-2), which were also associated to this group (Table 1, Figure 1). Group B consists of the peptide sequence LAFYACLAFC, found in a hypothetical protein from *Pseudomonas* bacteria with unknown function. This group also includes peptide M40 (LAFYACLAF) lacking the C-terminal cysteine residue of M14 and was designed with the aim to reduce the number of cysteines. To remove all cysteines from this peptide, M219 (LAFYASLAF) was designed containing a serine instead of cysteine at position 6 (Figure 1). The peptides M214 and M212 are both newly designed peptides based on the I24 sequence with similarities to peptides from bacterial proteins sharing two LAFYA motifs, with M214 containing one C-terminal cysteine and M212 containing a total of three cysteine residues (Table 1, Figure 1). Group C represents sequences with lower similarities to I24 at the primary sequence level but share the flanking cysteine residues and similar types of amino-acid residues. Group D contains a sequence (De1) from a phage surface display reported by Deng et al. [32] and a sequence derived from residues 507–516 of the bacteriophage T7 DNA-dependent RNA polymerase (T7RNAP) with lower similarities to I24 at the primary sequence level and lacking at least one terminal cysteine residue (Figure 1, Table 1).

### 2.2. Antiviral Activities of TAT-Fusion Peptides

For execution of its antiviral activity, the peptide I24 requires conjugation to a cell-penetrating peptide, with the TAT peptide as fusion partner showing strongest efficacy [25]. Therefore, all peptides were synthesized as fusion with the N-terminal TAT peptide (residues 48–60), except TAT-M214 and TAT-M212, which contain a TAT-Tag shortened by two amino-acids (residues 48–58) to reduce the overall size of the peptide. All TAT-fusion peptides were tested in two cell-based models we developed to study I24 [25] for their antiviral effects, i.e., baculovirus transduction of HEK293 cells and infection of NIH/3T3 cells with mouse cytomegalovirus (MCMV). Both indicator viruses express firefly luciferase, which was detected 24 h after transduction of HEK293 cells with baculovirus or 72 h after infection of NIH/3T3 cells with MCMV [33]. In addition, all peptides were tested for potential cell toxicity in HEK293 cells.

Table 2 shows the antiviral activity of the peptides in HEK293 cells transduced with baculovirus expressing luciferase, sorted by IC_50_ values in ascending order. The original peptide TAT-I24 was most potent, followed by TAT-T7 from group D. The peptide TAT-De1 from the same alignment group also showed antiviral activity, albeit slightly lower than TAT-I24 and TAT-T7 (Table 2). Antiviral activity was also observed with TAT-M40, TAT-M214 and TAT-M14 in the rank order of TAT-M40 > TAT-M214 > TAT-M14 for baculovirus-transduced HEK293 cells. Replacement of the single cysteine residue of TAT-M40 by serine, as in peptide TAT-M219, caused a reduction of antiviral potency (Table 2). From the peptides of group A containing peptide sequences found in the Nsp14 protein of beta-coronaviruses, TAT-M13 had best activity, but slightly lower compared to TAT-I24. The other peptides M17 and M22 from this group showed reduced antiviral activities. Peptides TAT-M41, TAT-M42 and TAT-43 from group C representing sequence motifs more unrelated to TAT-I24 had approximately 20-fold higher IC_50_ values in baculovirus-transduced HEK293 cells (Table 2).

Interestingly, peptide TAT-M212, which contains a duplication of the CLAFYAC motif from TAT-I24, had greatly reduced activity compared to TAT-I24, with an approximately 40-fold higher IC_50_ value in baculovirus-transduced HEK293 cells (Table 2). Similar results were previously reported by us with a peptide cyclized via the two cysteine residues (CLAFYACF; C22del_cyc), indicating that this shortened motif alone is not sufficient to cause an antiviral effect [33]. However, TAT-M214, which only has one C-terminal cysteine residue, showed potent antiviral activity, indicating that the additional cysteines in TAT-M212 may cause a structural constraint which impairs its antiviral potency.

Similar antiviral efficacies were obtained with NIH/3T3 cells infected with MCMV but with generally higher IC_50_ values previously reported for this system [25,33]. While both TAT-I24 and TAT-T7 were most potent, the peptides TAT-M13 and TAT-De1 also exerted antiviral activity. The designed peptides from group B showed antiviral activities in the rank order TAT-M40 > TAT-M14 > TAT-M214 for MCMV-infected NIH/3T3 cells. The peptide TAT-M219, which has a replacement of a single cysteine by serine of sequence TAT-M40, had greatly reduced activity in MCMV-infected NIH/3T3 cells (Table 2). The coronavirus-derived peptide sequences TAT-M17 and TAT-M22 also showed greatly reduced antiviral activity against MCMV while TAT-M13 had antiviral activity similar to TAT-I24. The peptide TAT-M212 with the duplicated motif CLAFYAC was much less active against MCMV infection of NIH/3T3 cells with a 20-fold higher IC_50_ value than TAT-I24. Due to their strongly reduced activity against baculovirus in HEK293 cells, the peptides TAT-M41, TAT-M42 and TAT-M43 were not included in further testing against MCMV (Table 2).

All peptides were also tested for potential cytotoxicity in HEK293 cells. The selectivity index (SI) for the transduction assay representing the ratio between antiviral activity and cytotoxicity (CC_50_/IC_50_) was calculated and is shown in Table 2. For some peptides, a partial reduction in cell viability was observed at higher concentrations with TAT-M219 exhibiting the lowest SI value (Table 2).

### 2.3. Effect of TAT-Fusion Peptides on m123/ie-1 Transcript Levels and pM123/IE1 Protein Expression in NIH/3T3 Cells

**Figure 2:**
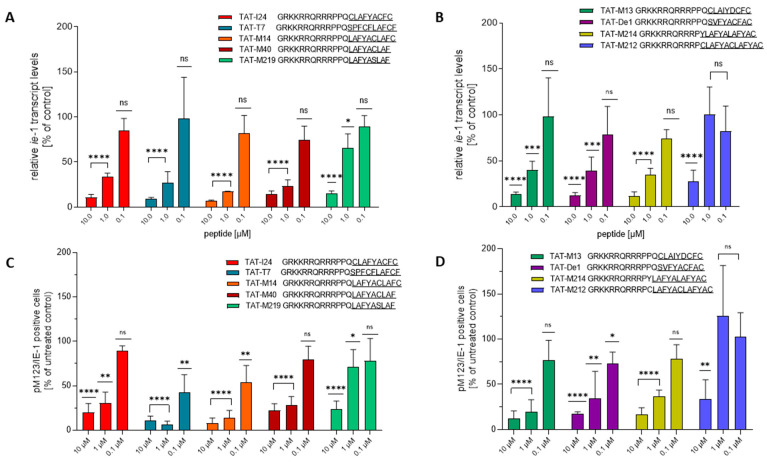
Effect of selected peptides on *m123/ie-1* transcript levels and pM123/IE1 protein expression. (**A**,**B**) NIH/3T3 cells were seeded into 48-well plates and treated on the next day with three different concentrations of peptides (10 µM, 1 µM, 0.1 µM) followed by infection with MCMV-Luc at a MOI 1 for two hours before isolation of total RNA and subsequent DNase digestion. Levels of *m123/ie-1* transcript levels relative to GADPH transcript levels (mean ± SD) were determined from cDNA by qPCR from three RNA preparations per group. (**C**,**D**) NIH/3T3 cells were seeded into ibidi slides and treated with three different concentrations of peptides (10 µM, 1 µM, 0.1 µM) and infected with MCMV-Luc at a MOI 1. Six hours after infection, cells were fixed and stained for pM123/IE1 and analyzed using a 20× objective. The number of pM123/IE1-postive cells relative to DAPI-stained cells was calculated and normalized to cells infected in the absence of peptide from three different locations of the slides and two independent experiments (results shown are mean ± SD). Image analysis was performed using ImageJ. Multiple *t*-test was used for statistical analysis: ns, represents statistically not significant at *p* ≥ 0.05, * represents statistically significant at *p* ≤ 0.05; ** represents statistically significant at *p* ≤ 0.01; *** represents statistically significant at *p* ≤ 0.001; **** represents statistically significant at *p* ≤ 0.0001.

In a previous study evaluating the effect of TAT-I24 peptide variants, their potency was analyzed in MCMV-infected NIH/3T3 cells by investigating their effect on *m123/ie-1* major immediate early transcript levels as well as the number of pM123/IE1-positive cells, which is produced by this transcript [33]. Selected peptides from the present study were also analyzed for these parameters. The peptides TAT-M17, TAT-M22, TAT-M41, TAT-M42 and TAT-M43 were not included in the testing due to their lower antiviral potencies against MCMV in NIH/3T3 cells. Apart from TAT-M219, which showed reduced potency to inhibit *m123/ie-1* transcript levels at 1 µM, a significant reduction of *m123/ie-1* transcript levels determined two hours post-infection was observed for all peptides at 10 and 1 µM (Figure 2A). The only exception was TAT-M212, which was unable to inhibit *m123/ie-1* transcript levels significantly at 1 µM (Figure 2B). The lower activities for peptides TAT-M219 and TAT-M212 are in accordance with their reduced efficacies on luciferase levels in MCMV-infected NIH/3T3 cells (Table 2).

In parallel, the percentage of pM123/IE1-postive NIH/3T3 cells six hours after infection with MCMV was determined by immunofluorescence staining as previously described [33]. Again, peptides TAT-M219 and TAT-M212, which were less active in the reporter gene assays (Table 2), also caused less reduction in the percentage of pM123/IE1 positive cells (Figure 2C,D).

### 2.4. Effect of TAT-Fusion Peptides on Entry of a DiO-Labelled MCMV

We have previously shown that TAT-I24 interferes with the early steps of MCMV virus entry. MCMV virions visualized upon their labelling with Vybrant™ DiO cell-labelling solution demonstrated an altered localization in the presence of TAT-I24 [33]. Cells infected with DiO-MCMV in the absence of TAT-I24 showed perinuclear organization of DiO-labelled virus two hours after infection, while a less organized and more dispersed localization of DiO-labelled virus particles is observed in the presence of TAT-I24 [33]. We applied this approach to the new TAT-fusion peptides from the present study. All active peptides showed similar effects as TAT-I24 on the localization of DiO-MCMV, indicating a very similar mode-of-action (Supplementary Figure S1).

**Figure 3:**
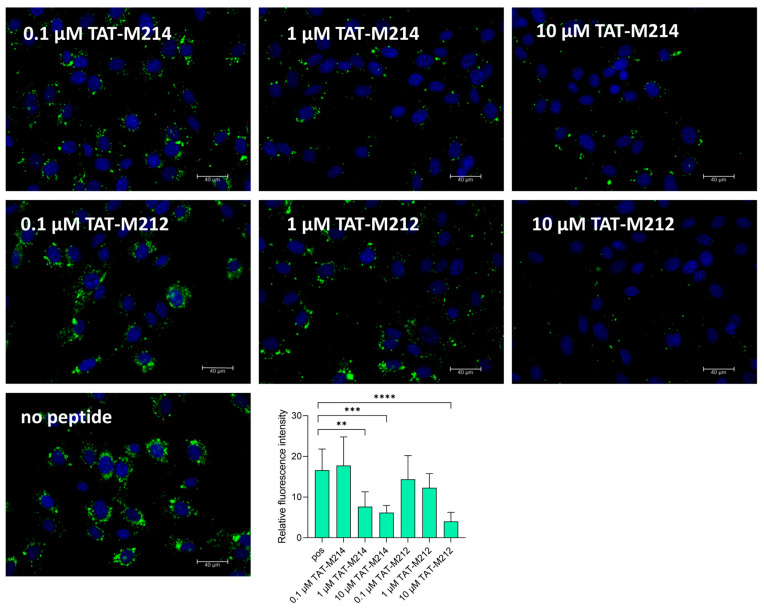
Localization of DiO-labelled MCMV in the absence or presence of different concentrations of peptides TAT-M214 and TAT-M212. NIH/3T3 cells were seeded into ibidi slides and infected on the next day with DiO-labelled MCMV-Luc at a MOI of 5. Two hours post-infection, cells were washed and fixed followed by staining with DAPI. Slides were then analyzed using a 40× objective. Images were taken from three different areas in each well; representative images from two independent experiments are shown. Relative fluorescence intensities relative to DAPI from three images from two independent experiments are shown (mean ± SD); ** represents statistically significant at *p* ≤ 0.01; *** represents statistically significant at *p* ≤ 0.001; **** represents statistically significant at *p* ≤ 0.0001.

In our previous study we demonstrated that TAT-I24 with a cyclized I24 peptide (CLAFYACF; C22del_cyc) shows greatly reduced antiviral activity and has no effect on localization of DiO-labelled MCMV [33]. As the peptide TAT-M212 representing two CLAFYAC motifs showed strongly reduced antiviral activity, we hypothesized a similar effect as with C22del_cyc. Indeed, TAT-M212 was able to affect perinuclear localization of DiO-labelled MCMV at 10 µM only, while at lower concentrations of 1 and 0.1 µM, a clear perinuclear organization could be seen (Figure 3). In contrast, the peptide TAT-M214, which exerts full antiviral activity, clearly affected correct localization of DiO-labelled MCMV at 1 and 10 µM (Figure 3).

### 2.5. Effect of TAT-Fusion Peptides on Red Blood Cells

In our previous publication, we have described the effect of TAT-fusion peptides on membrane integrity by testing them with freshly isolated red blood cells (RBC). When peptides were diluted in phosphate-buffered saline (PBS) and added to the cells, peptide-dependent swelling of RBCs was visualized by formation of a larger pellet after centrifugation of the plates. This effect was, however, not observed when peptides were diluted in the presence of 10% human serum, indicating that serum has a protecting effect possibly through binding of the peptides to serum components [33].

**Figure 4:**
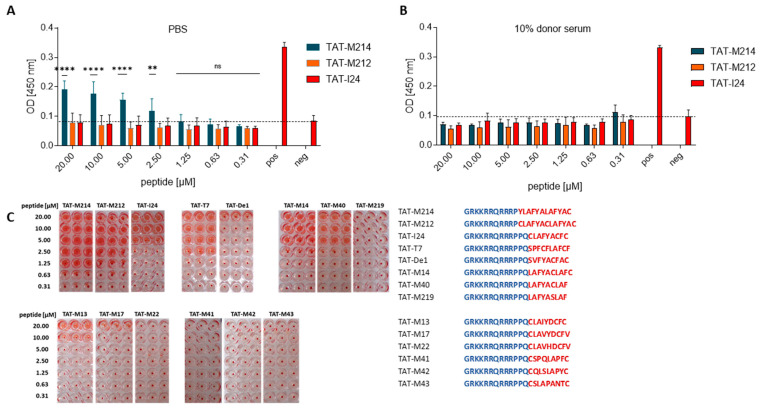
Effect of peptides on haemolysis and RBC swelling. (**A**,**B**) RBCs were isolated and treated in conical 96-well plates with peptides diluted in PBS or in 10% donor serum for one hour. OD [450 nm] was determined in the supernatants after centrifugation of the plates. Mean ± SD from two experiments in triplicates are shown. Multiple *t*-test was used for statistical analysis: **** represents statistically significant induction of haemolysis at *p* ≤ 0.0001, ** statistically significant induction of haemolysis at *p* ≤ 0.01; ns represents statistically not significant at *p* ≥ 0.05. Dashed lines indicate background OD. (**C**) Swelling of RBC after treatment with dilutions of peptides in PBS for one hour and centrifugation of the plates. Red wells indicate an enlarged cell pellet due to swelling of the RBCs. Sequences of the peptides are shown at the right and are indicated in red with the TAT fusion partner indicated in blue.

Next, we investigated the effect of the peptides from the present study on RBC swelling and haemolysis. Some peptides caused RBC swelling when the incubation was performed in PBS, but was not seen when peptides were applied in the presence of 10% human serum. In general, no or only very mild haemolysis was seen (Supplementary Figure S2). However, one exception was peptide TAT-M214, which induced haemolysis by 43% at 20 µM, 37% at 10 µM, 29% at 5 µM and 14% at 2.5 µM. This occurred only when the peptide was diluted in PBS and not in the presence of 10% serum (Figure 4A,B).

For most peptides, there was a clear connection between RBC swelling and antiviral activity, as peptides with strong antiviral activity (TAT-I24, TAT-T7, TAT-M13, TAT-M40, TAT-M14, TAT-M214) caused RBC swelling at higher concentrations as seen by larger pellets in photographic images of the plates after centrifugation, while peptides with lower antiviral activity, such as TAT-M219, TAT-M17, TAT-M22, TAT-M41, TAT-M42 and TAT-M43 also did not cause RBC swelling in the absence of serum even at 20 µM (Figure 4C). However, this rule does not strictly apply to all peptides, as TAT-De1 from group D, which showed antiviral activity, had less effect on RBC swelling, and TAT-M212, which had strongly reduced antiviral activity, caused RBC swelling over a broad concentration range comparable to TAT-M214 (Figure 4C). This demonstrates that it is possible to modulate antiviral activity and effects on membrane integrity by changes in the peptide sequence.

### 2.6. DNA-Binding of TAT-Fusion Peptides

**Figure 5:**
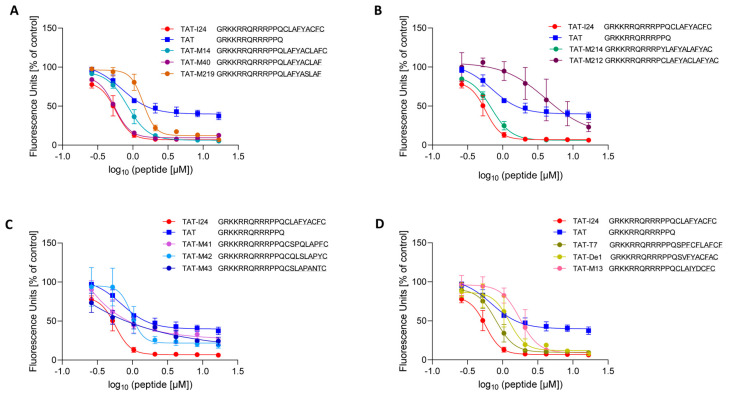
DNA-binding capacities of the peptides. Peptides were incubated with plasmid-DNA in the presence of SYBR^TM^ Gold dye. Reduction in fluorescence indicates binding of peptides to DNA. Results shown are DNA-binding of peptides TAT-I24, TAT, TAT-M14, TAT-M40 and TAT-M219 (**A**), of peptides TAT-I24, TAT, TAT-M214 and TAT-M212 (**B**), of peptides TAT-I24, TAT, TAT-M41, TAT-M42 and TAT-M43 (**C**), and of peptides TAT-I24, TAT, TAT-T7, TAT-De1 and TAT-M13 (**D**) and represent mean ± SD from three independent experiments performed in duplicates. Curve fitting was performed using GraphPad Prism software.

Since TAT-I24 binds dsDNA with high affinity, the TAT-fusion peptides from the present study were analyzed for their dsDNA-binding capacity using a fluorescence-based assay with TAT-I24 included as a reference [26]. We also included the TAT peptide, which binds DNA [34,35]. The TAT peptide also causes a decrease in fluorescence intensity but to a lower extent compared to TAT-I24, due to its lower affinity compared to TAT-I24, as previously shown [26]. The peptides with antiviral activity, such as TAT-M14 and TAT-M40 were found to bind dsDNA, while the peptide TAT-M219, which does not contain a cysteine residue, exhibited lower antiviral and DNA-binding capacities (Figure 5A). A similar observation was made for peptide TAT-M214 with marked antiviral activity and TAT-M212 with greatly reduced antiviral activity. These peptides also differed markedly in their binding to dsDNA, with TAT-M212 showing only weak DNA-binding (Figure 5B). The same applies to peptides TAT-M41, TAT-M42 and TAT-M43 with strongly reduced antiviral activity which also exhibited less dsDNA-binding (Figure 5C). One exception to this rule were peptides TAT-M13, TAT-T7 and TAT-De1, which showed reduced dsDNA-binding compared to TAT-I24, but were fully active in the antiviral assays (Figure 5D).

### 2.7. Structural Features of the TAT-Fusion Peptides

To identify common structural features of the peptides which could explain similarities or differences in their antiviral activities and dsDNA-binding capacities, a structure prediction was performed with all TAT-fusion peptides using the PEP-FOLD4 prediction tool [36,37,38,39,40]. The best models of TAT-I24 generated by the PEP-FOLD4 prediction tool show partial structural similarity with our previously reported model of the DNA-peptide complex [26]. Current PEP-FOLD4-derived models form two α-helical elements separated by a kink (Pro11–Pro12) between the TAT fusion partner and I24.

**Figure 6:**
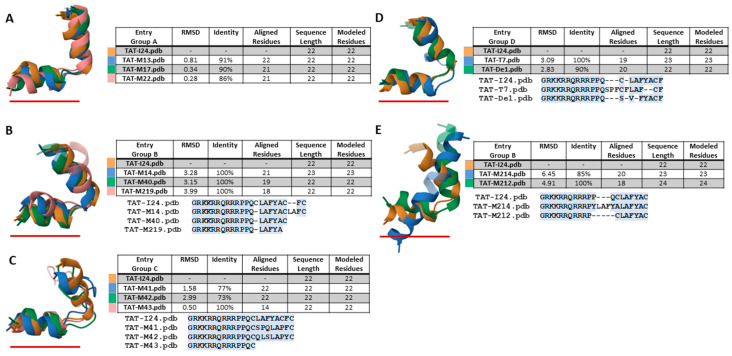
Structure prediction of TAT-fusion peptides and alignment of best models. Structure prediction was performed using the PEP-FOLD4 tool (http://bioserv.rpbs.univ-paris-diderot.fr/services/PEP-FOLD4 (accessed on 19 August 2025)). Best models were then structurally aligned to TAT-I24 using the alignment tool of the PDB protein data bank (Smith-Waterman; https://www.rcsb.org/alignment (accessed on 31 August 2025)). Alignment with TAT-I24 of peptides from group A (**A**), group B peptides TAT-M14, TATM40, TAT-M219 (**B**), peptides from group C (**C**), peptides from group D (**D**), and peptides TAT-M214 and TAT-M212 (**E**). The location of the TAT peptide in the structures is underlined in red. Results shown are aligned peptide models, RMSD and % identity over the aligned number of residues (indicated in blue).

The best models of all TAT-fusion peptides were then structurally aligned to the model-built TAT-I24 structure using the Protein Data Bank alignment tool (PDB) [41,42]. Alignment of peptide models from group A containing the peptide sequences derived from the Nsp14 protein of beta-coronaviruses (Figure 1) showed a general consensus of peptide structure over the entire peptide length, with low root mean square deviation values (RMSD) indicating a high structural similarity (Figure 6A). Superimposed TAT-fusion peptides of group B showed higher RMSD-values and less aligned amino-acid residues, in particular, TAT-M219, the least active peptide (Figure 6B). The peptides with lowest antiviral activities, TAT-M41, TAT-M42 and TAT-M43 showed least stable formations of an α-helical conformation of the part resembling peptides M41, M42 and M43, implying that a stable α-helical stretch at the C-terminal end may be an important feature of the antiviral effect. However, some structural similarities between TAT-I24 were found for TAT-M41 and TAT-M42, while TAT-M43 only aligned over the TAT part of the sequence and the first cysteine residue thereby resulting in a low RMSD-value (Figure 6C). Structural similarity was also observed for the predicted structures of TAT-I24, TAT-T7 and TAT-De1, albeit with larger deviations (Figure 6D). The peptides TAT-M214 and TAT-M212 with a shortened TAT-fusion partner showed a strong tendency to form a single stable α-helix over the entire peptide sequence. Consequently, the alignment of the best models with TAT-I24 suggests less structural compatibility due to the extended conformation of these peptides (Figure 6E). Although TAT-M214 exhibited antiviral activity, formation of a stable α-helix over the entire peptide sequence could lead to disturbance of membranes and could explain the enhanced effect of these peptides on RBC membrane swelling and haemolysis as with TAT-M214 (Figure 4A).

**Figure 7:**
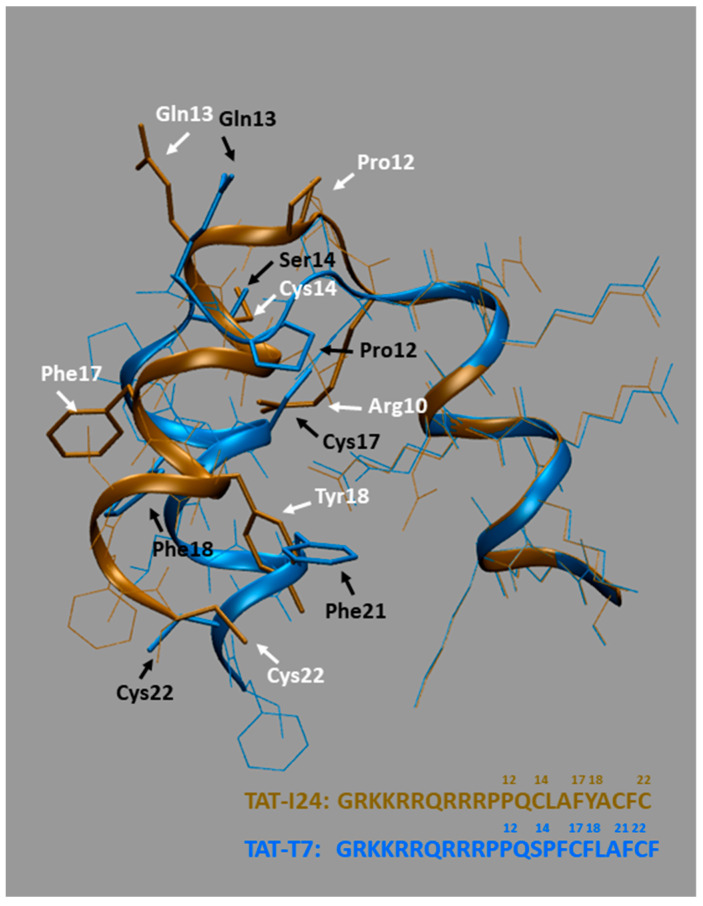
Superimposition of predicted structures of TAT-I24 and TAT-T7. Partial correspondences of amino-acid residues of TAT-I24 (brown) with TAT-T7 (blue) are shown by highlighted side-chains with positions indicated in white for TAT-I24 and in black for TAT-T7. Sequences and numbers of relevant residues are shown at the bottom.

However, although structural similarities were observed between the TAT fusion peptides, it remained open which residues of I24 and a peptide, such as T7 with low similarity at the primary amino-acid sequence level but comparable potency, could be responsible for the antiviral and DNA-binding effect. We therefore analyzed the superimposition of the predicted structures of TAT-I24 (in brown) and TAT-T7 (in blue) as shown in Figure 7. As expected, a perfect structural alignment was seen for residues Gly1 to Pro11 of the TAT-fusion partner with all positively charged side chains assuming orientations of maximal distance to each other due to electrostatic repulsion which makes them well suited for interaction with ds-RNA/DNA backbones [26,43]. However, Pro12 of TAT-T7 bends inwards, pointing almost into the opposite direction of Pro12 from TAT-I24, thereby aligning with Leu15 of TAT-I24, as seen with the highlighted Pro12 rings. Despite the different orientations of Pro12, the two following residues Gln13 and Cys14 (TAT-I24) and Ser14 (TAT-T7) still adopt comparable geometries. A rather strong structural deviation of Pro15 and Phe16 in TAT-T7 with no matching residues in TAT-I24 was observed. However, partial correspondences were found of Cys17 in TAT-T7 with Arg10 in TAT-I24, of Phe18 in TAT-T7 with Phe17 in TAT-I24 and of Phe21 in TAT-T7 with Tyr18 in TAT-I24, as shown by highlighted side-chains. No structural matches were identified for Leu19 and Ala20 in TAT-T7. Too much flexibility is assumed at the C-terminal end to identify precise structural matches but Cys22 could perhaps qualify.

## 3. Discussion

Mining of the human proteome to search for peptides with antimicrobial potential within internal protein segments led to the identification of novel antimicrobial peptides. Screenings were based on specific characteristics of such peptides including sequence length, net charge, average hydrophobicity as well as the cationic and amphiphilic nature [44,45]. Based on these properties of antimicrobial peptides, the software Kamal v 2.0 was developed to identify intragenomic antimicrobial peptides (IAP), encrypted in proteins encoded by plant and human genomes [46,47,48]. In our study, we utilized the primary sequence of the synthetic 9-mer peptide I24, part of the antiviral peptide TAT-I24 [25,26], to search for naturally occurring samples with sequence similarities to I24 to identify potential novel peptide sequences with improved nucleic acid-binding and antiviral activities. By performing Protein BLAST searches against known proteins, several peptide sequences could be identified which contain similar types of amino acids. The identified peptides were then fused to the TAT peptide and tested for antiviral activity in cell-based models and DNA-binding. In addition, structure prediction of the peptides and their alignment with TAT-I24 was performed to gain more insight into possible features required for the antiviral activity of the peptides.

The highest match with I24 at the primary sequence level was obtained with a sequence (LAFYACF) present in ORF56, the primase subunit of the WMHV and MuHV4 helicase-primase complex [49,50]. This heterotrimeric complex is the orthologue of the Human Herpesvirus-4 (HHV-4, EBV) helicase/primase (BSLF1, BBLF2/3 and BBLF4), which is essential for replication of the viral DNA [51,52]. However, a specific function of BSLF1, which is the homologue of ORF56, the subunit containing the I24-like sequence (amino acids 279–285), and its role in the interaction with DNA is currently not known.

A close similarity of I24 was also observed with peptide sequence 2N18 (SVFYACFACF), which was identified in the second round of a phage display screening against the E2 protein of human papillomavirus type 11 [32]. This peptide contains the motif CF/LXC, which was also identified in other peptide sequences obtained by several rounds of panning [32]. However, in their paper, only the result from the second panning round is shown for 2N18 and this peptide was not further experimentally verified [32]. Protein BLAST searches revealed that parts of similar sequences can be found in bacterial DEAD/DEAH box helicases. Homologous motifs were also found in the PDDEXK_9 nuclease domain of bacterial ATP binding proteins [53,54]. To investigate peptide 2N18 in more detail, a TAT-fusion peptide with this sequence only lacking the C-terminal phenylalanine (TAT-De1; SVFYACFAC) was generated and tested for antiviral activity. Indeed, TAT-De1 also exhibited antiviral activity, albeit slightly lower than TAT-I24, as was its DNA-binding capacity. One difference to I24 is the presence of an additional alanine between cysteines 6 and 9 of De1.

Another strong match using Protein BLAST was obtained with peptide M13 (CLAIYDCFC), a sequence found in the Nsp14 protein of the beta-coronavirus HKU1 [55]. A further homology-based search of the M13 sequence was performed against other beta-coronaviruses. The peptides CLAVYDCFV (M17) from Nsp14 of bat beta-coronavirus and CLAVHDCFV (M22) from SARS-CoV-2 were selected for further investigations. The Nsp14 protein belongs to the non-structural proteins of beta-coronaviruses and is a bifunctional protein, consisting of an N-terminal 3′-to-5′ exoribonuclease (ExoN) and a C-terminal N7-methyltransferase (N7-MTase) domain, and plays a role in RNA proofreading by removing mismatched nucleotides in a metal ion dependent manner [56]. The peptide sequence used in the present study is located at the C-terminal end of the ExoN domain of Nsp14 (amino acids 276–284), close to the hinge region [57] and the crystal structure shows a zinc atom binding to the first cysteine residue of this peptide sequence [58]. However, the exact functionality of these positions in Nsp14 remains to be elucidated. A peptide fusion of M13 to the TAT-peptide (TAT-M13) indeed caused antiviral activity in two cell-based systems, albeit slightly lower compared to TAT-I24. The two other peptides M17 and M22 were also tested for antiviral activity as TAT-fusion peptides. Both peptides exhibited some antiviral activity but to a much lower extent than TAT-I24 or TAT-M13.

In our previous study describing the DNA-binding properties of I24, blocking of T7-RNA polymerase-dependent RNA (T7RNAP) synthesis by I24 was observed [26]. We therefore analyzed the protein sequence of this enzyme and identified a putative interesting peptide sequence, SPFCFLAFCF, located at positions 507–516 of T7RNAP within the palm insertion module (amino acids 450–527) of the polymerase domain. The palm insertion module of T7RNAP containing this sequence has been described to close the putative nucleic acid-binding channel, but the exact functionality of this domain in not known yet [59,60]. However, the crystal structure did not indicate that this peptide sequence is close to the dsDNA of the T7 promoter [61] and it remains to be elucidated whether this region interferes directly with DNA. The sequence identified in T7RNAP has lower homology with I24 at the level of the primary sequence, but contains similar motifs and structural features, including the LAF motif and two cysteine residues. Indeed, fusion of T7 to the TAT peptide generated a peptide with strong antiviral activity as well as DNA-binding activity. As with TAT-I24, TAT-T7 also caused RBC swelling when added in PBS but not in the presence of donor serum. Superimposition of the predicted structures indicated partial correspondences of specific amino acids of TAT-I24 and TAT-T7 involving cysteine residues and amino acids with aromatic side-chains. However, a more detailed experimental analysis would be required to confirm the importance of these residues in TAT-T7 for its antiviral effect.

Another candidate peptide with the sequence LAFYACLAFC (M14) was found from a hypothetical protein in *Pseudomonas* bacteria. It contains the LAF motif twice, which may enhance the activity of the peptide. As TAT-fusion peptide, TAT-M14 exhibited antiviral activity and DNA-binding capacity. A shorter version, M40 (LAFYACLAF) even showed improved antiviral and DNA-binding activity as TAT-fusion peptide. To investigate the requirement of the single cysteine residue for its activity, peptide M219 (LAFYASLAF) was designed and synthesized as TAT-fusion peptide. Replacement of cysteine by serine in this peptide led to a strong reduction in antiviral activity against MCMV in NIH/3T3 cells and greatly reduced DNA-binding. This confirms previous observations that free cysteine residues are important for antiviral activity [33].

It has been reported that the TAT peptide can cause an antiviral effect when a C-terminal free cysteine residue is added [62]. It is possible that the free cysteine causes a stabilizing effect by attaching the peptide to serum components such as albumin, which contains a free cysteine [63,64]. We therefore investigated whether peptides with lower homologies to I24 but containing flanking cysteine residues may also exert an antiviral activity. Three peptides, CSPQLAPFC (M41), CQLSLAPYC (M42) and CSLAPANTC (M43) were selected, based on earlier database searches. Fusion of these peptides to the TAT peptide and testing in the HEK293 baculovirus transduction assay showed greatly reduced antiviral activities. They also showed DNA-binding activity more similar to the TAT peptide alone, demonstrating that a peptide sequence containing free cysteine residues alone is not sufficient for exerting antiviral activity. Simulation of the peptide structure showed that peptides in the C-terminal part of the TAT-fusion peptide, M41, M42 and M43, formed unordered structures, which is in in contrast to the formation of stable α-helices by antivirally active peptides, indicating that there are specific structural requirements for the antiviral activity. Interestingly, these peptides also did not cause RBC swelling nor haemolysis, which they have in common with the TAT peptide, or less active peptides from a previous study [33].

Aiming to identify more active peptides, another TAT-fusion peptide was generated of the sequence YLAFYALAFYAC (M214) containing a repeat of the LAFYA motif with a shortened TAT peptide (residues 48–58). TAT-M214 was active in the antiviral assay using HEK293 baculovirus transduction but less in MCMV-infected NIH/3T3 cells. It also exhibited DNA-binding activity comparable to TAT-I24. However, this peptide had strong effects on RBC swelling and caused haemolysis when applied in PBS. Structure prediction indicates formation of a rigid α-helix without bending as predicted for the other antiviral active peptides, which can be explained by the absence of the second proline residue in the TAT part of the peptide. It is possible that this helical structure inserts into the cell membranes and can lead to damaging effects, as witnessed by its induction of haemolysis. Interestingly, a version of this peptide, M212 (CLAFYACLAFYAC), which consists of a duplication of the CLAFYAC motif, was assumed to fulfil all features thought to be required for an antiviral peptide. However, the peptide TAT-M212 did not exert antiviral activity, and this was observed with two different batches of peptides excluding a synthesis-dependent effect. Nevertheless, the peptide caused RBC swelling, indicating that effects on membranes observed for antiviral peptides are not a pre-requisite for antiviral activity.

Structure prediction of the peptides indicated a helical hairpin motif combining the TAT fusion partner with the I24 part. Similarities in the predicted structures were found with all the peptides tested, except for the peptides with lower similarity to TAT-I24, such as TAT-M41, TAT-M42 and TAT-M43, which were predicted to form less stable α-helical conformations. However, due to structural dynamics, peptides could adopt different conformations depending on the environment, such as in the presence of cellular membranes [24]. Experimental verification of such structures by circular dichroism or nuclear magnetic resonance (NMR) would be required [65,66].

Together, we have identified novel antiviral peptides based on the prototypic sequence TAT-I24, which demonstrates that similar peptides can reproduce its antiviral effect with the same mode-of-action. Of note is that these sequences were frequently found in proteins interacting with nucleic acids, such as T7RNAP, primases and helicases as well as the ExoN domain of Nsp14 of beta-coronaviruses. However, with all these proteins, no specific function could be assigned yet to the regions containing these specific sequences. Thus, further extensive mechanistic studies for each of the proteins would be required.

Although the virus infection models employed in the presented study are very useful for selecting novel candidates, further testing against other dsDNA-viruses [25] may identify peptides with superior efficacy in a particular virus model. Based on their activity profiles, TAT-T7, TAT-M40 and TAT-De1 are certainly candidates which will be further investigated. However, from the mechanistic perspective, one major challenge when aiming at a broad-acting candidate is the difference in the entry pathways of viruses or mutations in the virus genome which could affect the mode of virus entry. One limitation is the dependence of the sensitivity to the TAT-fusion peptides on the viral entry pathway as described for SARS-CoV-2 variants [27] and adenovirus serotypes [25]. Therefore, future research will also focus on the adaption of the fusion partner to achieve peptides with an even broader target profile. Apart from these mechanistic challenges, additional hurdles in the development of peptides are the low systemic stability of peptides in general, peptide aggregation as well as cost-intensive peptide synthesis [5]. Chemical modifications that enhance peptide stability, combined with the evaluation of bacterial expression systems for peptide biosynthesis, may overcome existing limitations and thereby expand the therapeutic potential of peptides as antiviral agents. The recent approval of Bulevirtide (Hepcludex^®^), a synthetic N-acylated 47-mer peptide derived from the hepatitis B virus (HBV) envelope protein that has been shown to block viral entry, confirms the innovative therapeutic potential of antiviral peptides and their successful entry into clinical practice as a new class of substances for treating virus infections [67].

## 4. Materials and Methods

### 4.1. Peptides

TAT-I24 and TAT have been described previously [68]. Peptides TAT-M14, TAT-M214, TAT-M212 and TAT-M219 were synthesized at Synpeptide Co., Ltd. (Shanghai, China). The peptides TAT-M13, TAT-T7, TAT-De1, TAT-M40, TAT-M17, TAT-M22, TAT-M41, TAT-M42 and TAT-M43 were synthesized at ProteoGenix (Schiltigheim, France). Purities were determined by high-performance liquid chromatography and mass spectrometry (Supplementary Table S1). All peptides were dissolved in dimethyl sulphoxide (Merck, Schnelldorf, Germany) at a concentration of 10 mM and stored in aliquots at −20 °C.

### 4.2. Cell Culture

HEK293 and NIH/3T3 cells were grown in a CO_2_-independent medium containing 10% FCS, 2 mM glutamine and 1% antibiotic-antimycotic (ThermoFisher, Darmstadt, Germany) and cultivated in a humidified atmosphere at 37 °C. Cells were passaged once a week.

### 4.3. Viruses

#### 4.3.1. Baculovirus

A baculovirus expressing luciferase under the control of the CMV promoter has been described previously [25]. For assessing the antiviral activity, HEK293 cells were seeded at a density of 2 × 10^4^ cells/100 µL per well of 96-well plates (Sarstedt, Nümbrecht, Germany). On the next day, cells were treated with 1:3 dilution series of peptides starting at a concentration of 20 µM followed by transduction with Baculovirus-Luc at a multiplicity of infection (MOI) of 5. All treatments were performed in triplicates. After 24 h, cells were lysed using 20 µL Cell Culture Lysis Reagent (Promega, Mannheim, Germany). Luciferase activity was recorded from 10 µL of the lysates using Luciferase Assay System and a GloMax^®^-Multi Detection System (Promega, Mannheim, Germany), as described previously [33].

#### 4.3.2. Mouse Cytomegalovirus

NIH/3T3 cells were seeded at a density of 2 × 10^4^ cells/100 µL per well of a 96-well plate (Sarstedt, Nümbrecht, Germany) and allowed to attach overnight. On the next day, cells were treated with 1:3 dilutions series of peptides starting at a concentration of 20 µM followed by infection with MCMV strain delm157-luc rep [69] at a MOI of 0.5 with the aid of centrifugal enhancement twice at 800× *g* for 15 min at room temperature. All treatments were performed in triplicates. Cells were lysed 72 h post-infection using 20 µL Cell Culture Lysis Reagent (Promega, Mannheim, Germany). Luciferase activity was recorded from 10 µL of the lysates using Luciferase Assay System and a GloMax^®^-Multi Detection System (Promega, Mannheim, Germany), as described previously [33].

### 4.4. Cell Viability Assay

HEK293 cells were seeded at a density of 2.5 × 10^3^ cells/100 µL per well of a 96-well plate (Sarstedt, Nümbrecht, Germany). On the next day, cells were treated with serial 1:3 peptide dilutions starting with a concentration of 20 µM in triplicates and incubated for 72 h at 37 °C. Then, 20 µL of CellTiter-Blue^®^ Cell Viability Assay (Promega, Mannheim, Germany) was added to each well and plates incubated at 37 °C for 1–2 h. Fluorescence was recorded using a GloMax^®^-Multi Detection System (Promega, Mannheim, Germany). The percentage of viable cells was calculated from the cell dilution series from each plate by linear regression analysis. The selectivity index was calculated using the following formula: SI = CC_50_/IC_50_, as described previously [33].

### 4.5. Staining and Microscopy

NIH/3T3 cells were seeded at a density of 2 × 10^4^ cells/250 µL per well of ibiTreat 8-well chambers (ibidi, Gräfelfing, Germany) and allowed to attach overnight at 37 °C. On the next day, cells were treated with peptides at concentrations of 10 µM, 1 µM and 0.1 µM and infected with MCMV at a MOI of 1 with the aid of centrifugal enhancement twice at 800× *g* for 15 min. Six hours post-infection, supernatants were removed and cells fixed with 5% formaldehyde for one hour at room temperature, followed by permeabilization with 0.5% Triton X-100 (Merck, Schnelldorf, Germany) in PBS for 10 min at room temperature. Blocking of the slides was performed using 3% bovine serum albumin (BSA; Merck, Schnelldorf, Germany) in PBS for one hour. Then, anti-m123/IE1 antibody (clone IE1.01; Center for Proteomics, Rijeka, Croatia) was added at a 1:400 dilution in PBS + 1% BSA and slides were incubated overnight at 4 °C. On the next day, wells were washed three times with PBS and goat anti-mouse IgG Texas Red (#T6390; (ThermoFisher, Darmstadt, Germany), diluted 1:100 in PBS + 1% BSA was added. Slides were incubated for one hour at room temperature. After another two washes with PBS, wells were incubated for 10 min with 1 µg/mL 4′,6-Diamidino-2-Phenylindole, Dihydrochloride (DAPI; ThermoFisher, Waltham, MA, USA). Image analysis was performed using ImageJ software v 1.54g. Red-positive cells were counted and normalized to DAPI-positive cells.

DiO-labelling MCMV was generated, as described previously [33]. For microscopy, NIH/3T3 cells were seeded at a density of 2 × 10^4^ cells/250 µL per well of ibiTreat 8-well chambers (ibidi, Gräfelfing, Germany) and incubated overnight at 37 °C. On the next day, cells were treated with peptides at concentrations of 10 µM, 1 µM and 0.1 µM and infected with DiO-labelled MCMV corresponding to a MOI of approximately 5 with the aid of centrifugal enhancement twice at 800× *g* for 15 min. After 2 h incubation, cells were washed twice with PBS and followed by fixation with 5% formaldehyde. Then, cells were stained with DAPI for 15 min, as described above. Cells stained for pM123/IE-1 were inspected using a 20× objective, and DiO-labelled cells using a 40 × oil objective of the Live Cell Video Microscope (Leica Microsystems, Wetzlar, Germany) provided by the BOKU Imaging Center (Vienna, Austria). Image analysis was performed using ImageJ software v 1.54g using the color threshold tool.

### 4.6. RNA Isolation, cDNA Synthesis and Quantitative Real-Time PCR

NIH/3T3 cells were seeded at a density of 4 × 10^4^ cells/250 µL per well of a 48-well plate and allowed to attach overnight at 37 °C. On the next day, cells were treated with peptides at concentrations of 10 µM, 1 µM and 0.1 µM and infected with MCMV at a MOI of 1 with the aid of centrifugal enhancement twice at 800× *g* for 15 min. Two hours post-infection, cells were lysed using RLT reagent (Qiagen, Hilden, Germany). RNA isolation, DNase digestion, cDNA reaction and qPCR were performed, as described previously [68].

### 4.7. Isolation of Red Blood Cells and Haemolysis Assay

Whole blood was collected from a healthy volunteer after informed consent by venipuncture into K2E K2EDTA VacuetteTM tubes (Greiner Bio-One, Kremsmünster, Austria) with 4 mL blood. The assay for haemolysis was adapted from Sæbo et al. [70] and performed as described previously [33]. In brief, blood was centrifuged at 1000× *g* for 10 min, and plasma kept for further use, followed by resuspension of red blood cells (RBC) in PBS and centrifugation for three times. RBC were seeded at a cell number of 1 × 10^7^ cells/100 µL PBS per well into conical 96-well plates (ThermoFisher, Darmstadt, Germany) and incubated with 100 µL of 1:2 peptide dilutions starting from 40 µM either in PBS or 10% donor serum. Plates were incubated at 37 °C for one hour before at 1000× *g* for 5 min. Supernatants were then transferred to flat 96-well plates and optical density (OD) was determined at 450 nm using a Glo-Max^®^-Multi Detection System (Promega, Mannheim, Germany). As positive control, cells were lysed with 0.5% Triton X-100. Images from centrifuged plates were taken using a Kodak PIXPRO camera (Rochester, NY, USA).

### 4.8. DNA-Binding Assay

DNA binding assay was performed, as described previously [26].

### 4.9. Structure Prediction

Structure prediction was performed using PEP-Fold4 prediction tool [36,37,38,39,40]. Alignment of structures was performed using PDB alignment tool using the Smith–Waterman algorithm [41]. Structural superpositions were downloaded from the PDB (https://www.rcsb.org/alignment (accessed on 31 August 2025)) and visually examined in VMD [71].

### 4.10. Database Searches

Protein BLAST analysis was performed through the NCBI home page at https://www.ncbi.nlm.nih.gov (accessed on 7 July 2025) [30,31].

### 4.11. Data Analysis

Statistical analysis was performed using GraphPad Prism 8 (GraphPad Software, San Diego, CA, USA). Protein alignments were performed using CLC Biobench 25 (Qiagen; https://digitalinsights.qiagen.com/, accessed on 2 November 2025). Image analysis was performed using ImageJ v 1.54g [72].

## 5. Patents

Hanna Harant is the inventor of patent application WO2019/057973 “Gene expression inhibitors”.

## Figures and Tables

**Table 1:** Peptide sequences identified by Protein BLAST using the I24 peptide sequence as query.

					Synthetic Peptide *	
Sequence BLAST Hit	ID	Species	Amino Acids	Protein Name/Function	Name	Sequence
LAFYACF	YP_010085930.1	Wood mouse herpesvirus (WMHV)	279–285	Helicase-primase primase subunit	I24	CLAFYACFC
	NP_044893.1	Murid gammaherpesvirus 4 (MuHV4)	279–285			
CLAIYDCFC	ABD75543.1	Human coronavirus HKU1	6325–6333 (ORF1ab) 276–284 (Nsp14)	Nonstructural protein 14/Guanine-N7 methyltransferase	M13	CLAIYDCFC
CLAVYDCFV	UUT43647.1	*Betacoronavirus* sp.	6317–6325 (ORF1ab) 276–284 (Nsp14)	Nonstructural protein 14/Guanine-N7 methyltransferase	M17	CLAVYDCFV
CLAVHDCFV	UPB65199.1	SARS-CoV-2	6204–6212 (ORF1ab) 275–283 (Nsp14)	Nonstructural protein 14/Guanine-N7 methyltransferase	M22	CLAVHDCFV
SVFYACFSC	MDI3365062.1	*Pantoea* sp. V108_6	567–575	DEAD/DEAH box helicase/RNA-binding proteins with ATPase activity	De1 **	SVFYACFAC
SVFYACFA	WP_008932954	*Ectothiorhodospira* sp. PHS-1	423–430	ATP-binding protein		
LAFYACLAFC	WP_240521350.1	*Pseudomonas*	140–149	Hypothetical protein	M14	LAFYACLAFC
SPQLAPFC	MFV1968217.1	Pirellulaceae bacterium	145–152	SDR family oxido-reductase/NAD- or NADP-dependent oxidoreductases	M41	CSPQLAPFC
QLSLAPYC	XP_060411117.1	Colletotrichum navitas	212–219	LY79DRAFT_318317/uncharacterized protein	M42	CQLSLAPYC
CSLAPANTC	MFA5187732.1	Patescibacteria group bacterium	819–827, 1015–1023	Repeated sequence motifs in Ig-like domain-containing protein	M43	CSLAPANTC
CSLAPTNTC			524–532, 722–730, 1113–1121			
SPFCFLAFCF	P00573	bacteriophage T7	507–516	T7 RNA Polymerase/DNA-directed RNA polymerase	T7	SPFCFLAFCF
LAFYALAFY	MBK9337318.1	Lewinellaceae bacterium	128–136	IPM98_12370/hypothetical protein	M214	YLAFYALAFYAC
LAFFACLAFY	XP_013439911.1	Eimeria necatrix	43–52	RER1 protein/Retrieval of endoplasmic reticulum membrane proteins from the early Golgi compartment	M212 ***	CLAFYACLAFYAC

* C-terminal part of TAT-fusion peptide (see Section 2.2), names and sequences of synthetic peptides highlighted by grey background. ** Based on partial sequence from 2N18 (SVFYACFACF) identified by phage display by Deng et al., 2004 [32]. *** Generated by rational design, contains duplication of CLAFYAC motif.

**Table 2:** Antiviral activity and cell toxicity of the peptides.

Name	Sequence	Group	Baculovirus/ HEK293 IC_50_ [µM]	SI [HEK293]	MCMV/ NIH3T3 IC_50_ [µM]
TAT-I24	GRKKRRQRRRPPQCLAFYACFC		0.016	2480 [33]	0.809
TAT-T7	GRKKRRQRRRPPQSPFCFLAFCF	D	0.017	1299	1.126
TAT-M40	GRKKRRQRRRPPQLAFYACLAF	B	0.021	2585	1.846
TAT-M214	GRKKRRQRRRPYLAFYALAFYAC	B	0.025	2306	3.668
TAT-M13	GRKKRRQRRRPPQCLAIYDCFC	A	0.031	1731	1.219
TAT-M14	GRKKRRQRRRPPQLAFYACLAFC	B	0.034	766	2.569
TAT-De1	GRKKRRQRRRPPQSVFYACFAC	D	0.037	517	1.611
TAT-M219	GRKKRRQRRRPPQLAFYASLAF	B	0.096	24	7.379
TAT-M17	GRKKRRQRRRPPQCLAVYDCFV	A	0.102	792	5.849
TAT-M22	GRKKRRQRRRPPQCLAVHDCFV	A	0.245	138	11.78
TAT-M43	GRKKRRQRRRPPQCSLAPANTC	C	0.306	n.d.	n.d.
TAT-M42	GRKKRRQRRRPPQCQLSLAPYC	C	0.378	n.d.	n.d.
TAT-M41	GRKKRRQRRRPPQCSPQLAPFC	C	0.434	n.d.	n.d.
TAT-M212	GRKKRRQRRRPCLAFYACLAFYAC	B	0.619	>10,000	18.190

n.d. not determined; SI Selectivity index (CC_50_/IC_50_).

## Data Availability

The raw data supporting the conclusions of this article will be made available by the authors on request. The original contributions presented in this study are included in the article/Supplementary Materials. Further inquiries can be directed to the corresponding author.

## Supplementary Materials

The following supporting information can be downloaded at: https://www.mdpi.com/article/10.3390/ijms262311433/s1.

## Abbreviations

The following abbreviations are used in this manuscript:

AMP	Antimicrobial peptide
RBC	Red blood cells
T7RNAP	T7 DNA-dependent RNA polymerase
PBS	phosphate-buffered saline
