# Supplementary material for: Identification of Novel TAT-I24-Related Peptides with Antiviral Activities
Source: Int J Mol Sci. 2025 Nov 26;26(23):11433. doi: 10.3390/ijms262311433 (PMC12692169; doi:10.3390/ijms262311433)
Supplement: Supplementary file 1 [file ijms-26-11433-s001.zip › ijms-3999558-supplementary.pdf]

Supplementary Figure S1

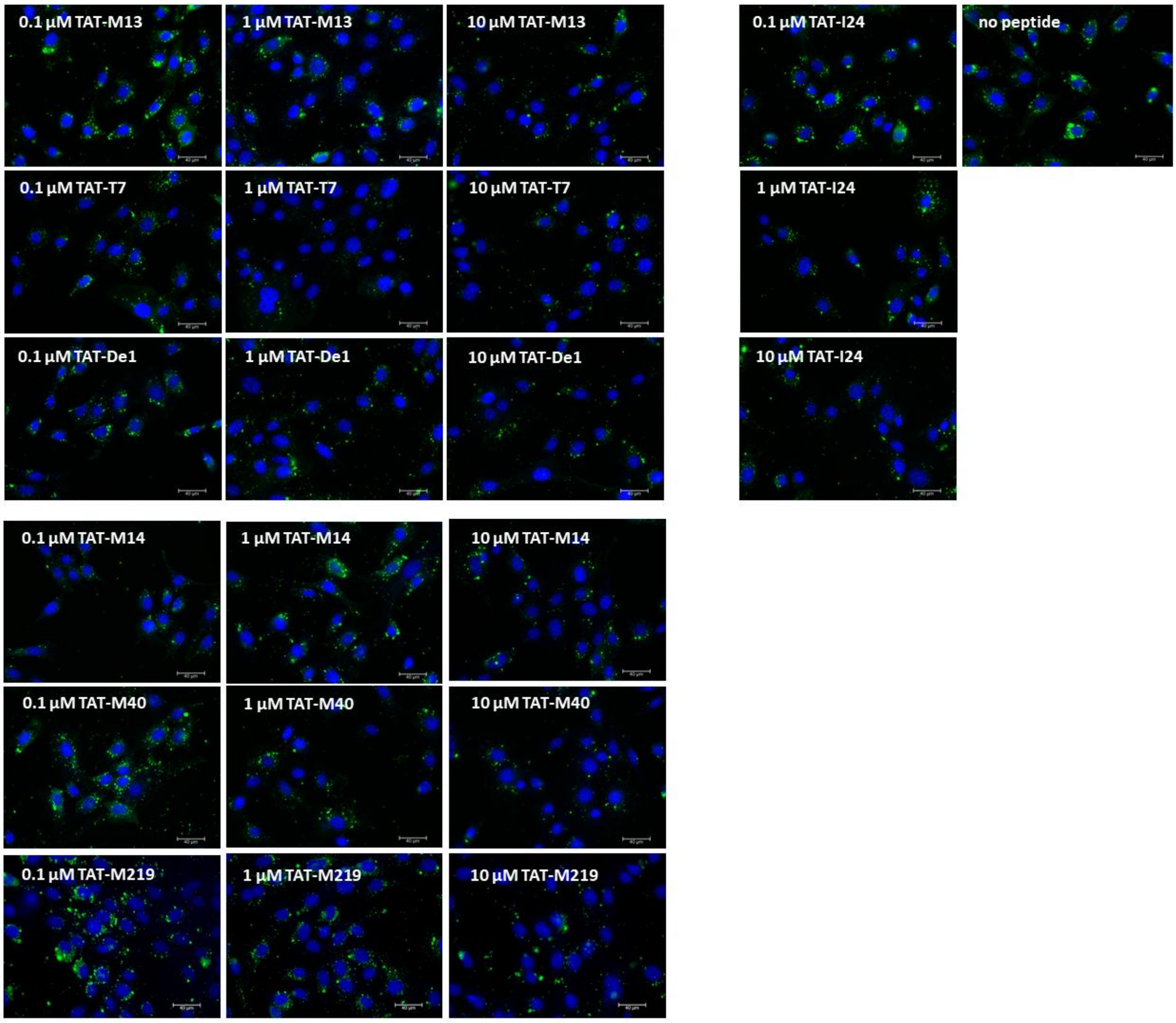

Supplementary Figure S1. Localization of DiO-labelled MCMV in the absence or presence of different concentrations of peptides TAT-M13, TAT-T7, TAT-De1, TAT-M14, TAT-M40, TAT-M219 and TAT-I24. NIH/3T3 cells were seeded into ibidi slides and infected on the next day with DiO-labelled MCMV-Luc at a MOI of 5. Two hours post-infection, cells were washed and fixed, followed by staining with DAPI. Slides were analyzed using a 40 × objective. Images were taken from three different areas in each well, representative images from two independent experiments are shown.

Supplementary Figure S2

A

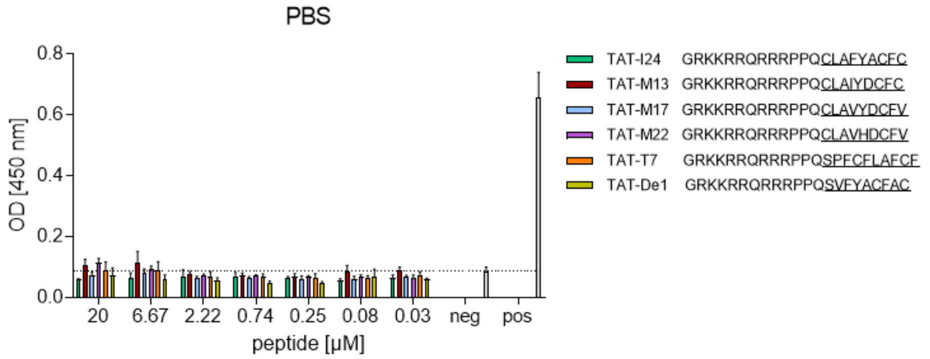

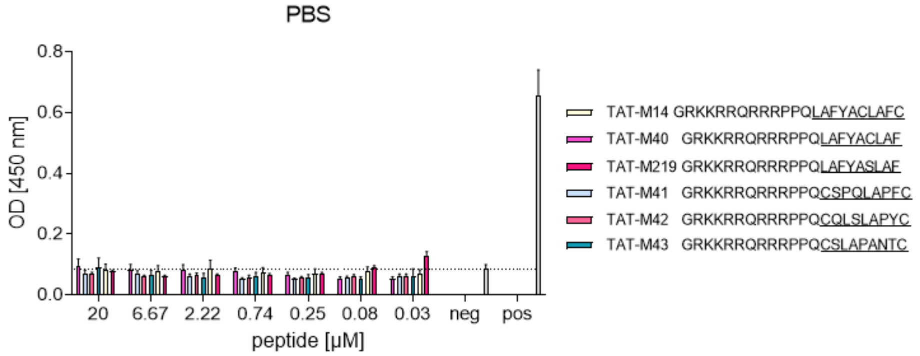

B

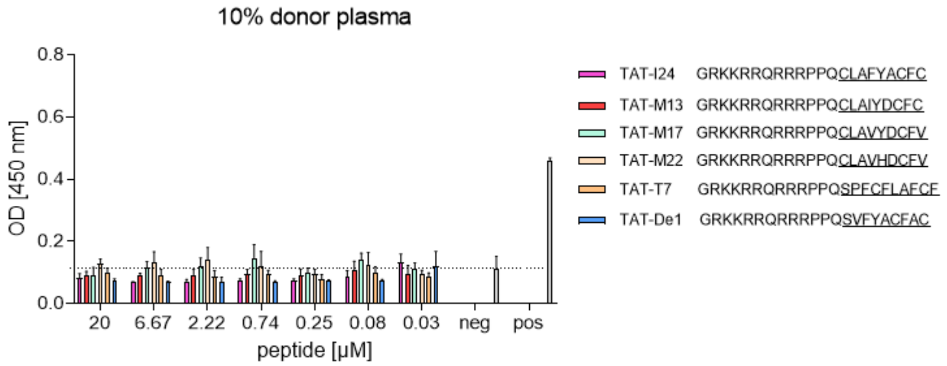

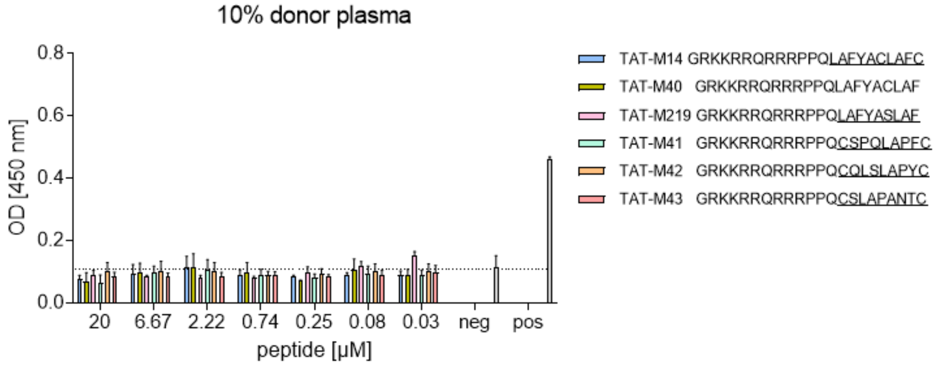

Supplementary Figure S2. No effect of peptides on hemolysis. RBC´s were isolated and treated in conical 96-well plates with peptides diluted in PBS (A) or in 10% donor serum (B) for one hour. OD [450 nm] was determined in the supernatants after centrifugation of the plates. As positive control, 0.5% Triton X-100 was used. Mean ± SD from two experiments in triplicates are shown.

Supplementary Table S1. Purities of the peptides used in the study

NAME	SEQUENCE	PURITY (HPLC)
**TAT-I24**	GRKKRRQRRRPPQCLAFYACFC	97.20%
**TAT-T7**	GRKKRRQRRRPPQSPFCFLAFCF	84.54%
**TAT-M40**	GRKKRRQRRRPPQLAFYACLAF	74.12%
**TAT-M214**	GRKKRRQRRRPYLAFYALAFYAC	95.64%
**TAT-M13**	GRKKRRQRRRPPQCLAIYDCFC	89.58%
**TAT-M14**	GRKKRRQRRRPPQLAFYACLAFC	95.20%
**TAT-De1**	GRKKRRQRRRPPQSVFYACFAC	80.11%
**TAT-M219**	GRKKRRQRRRPPQLAFYASLAF	95.45%
**TAT-M17**	GRKKRRQRRRPPQCLAVYDCFV	95.37%
**TAT-M22**	GRKKRRQRRRPPQCLAVHDCFV	91.21%
**TAT-M43**	GRKKRRQRRRPPQCSLAPANTC	92.61%
**TAT-M42**	GRKKRRQRRRPPQCQLSLAPYC	72.32%
**TAT-M41**	GRKKRRQRRRPPQCSPQLAPFC	77.24%
**TAT-M212**	GRKKRRQRRRPCLAFYACLAFYAC	95.40%
